# Supplementary material for: Diagnosis of Depth of Submucosal Invasion in Colorectal Cancer with AI Using Deep Learning
Source: Cancers (Basel). 2022 Oct 31;14(21):5361. doi: 10.3390/cancers14215361 (PMC9656054; doi:10.3390/cancers14215361)
Supplement: Supplementary file 1 [file cancers-14-05361-s001.zip › cancers-1954167-supplementary.pdf]

Supplementary Table S1. Clinicopathological features and statistical analysis between learning and validation sets.

Variables	Learning set (2011-2015)	Validation set (2016-2017)	P-Value
	n = 91	*n* = 49	
**Age, median (years) ***	66 (41–86)	66 (35–84)	0.740
**Sex (male/female)**	59/32	28/21	0.371
**Location (colon/rectum)**	54/37	32/17	0.489
**Greatest diameter, median (mm)**	20 (0–50)	20 (6–66)	0.703
**Preoperative CEA (≥5/5</NA) (ng/mL)**	12/78/1	11/36/2	0.362
**Preoperative CA19-9 (≥38/38</NA) (ng/mL)**	5/85/1	3/44/2	0.845
**Degree of differentiation (tub1/tub2/others)**	57/30/4	29/19/1	0.474
**Depth of invasion, median (μm) ***	2000 (0–9000)	2200 (91–12000)	0.324
**Lymph node metastasis (+/-)**	7/84	7/42	0.215
**Lymphatic invasion (+/-)**	24/67	16/33	0.433
**Vascular invasion (+/-)**	12/79	7/42	0.856
**Budding grade (1/2, 3/NA)**	54/16/21	37/8/4	0.513

* Continuous variables were evaluated.

CEA, Carcinoembryonic antigen; CA19-9, Carbohydrate antigen 19-9; NA, Not applicable

Supplementary Table S2. The percentage of endoscopic images with incorrect AI diagnosis in the validation set.

		**AI diagnosis**	
		Tumor area	Other area
**Pathological classification**	SM-s 64 images	28 images (43.8%)	36 images (56.2%)
	SM-d 38 images	15 images (39.5%)	23 images (60.5%)
